# Hyaluronic Acid Based Hydrogels for Regenerative Medicine Applications

**DOI:** 10.1155/2015/871218

**Published:** 2015-05-19

**Authors:** Assunta Borzacchiello, Luisa Russo, Birgitte M. Malle, Khadija Schwach-Abdellaoui, Luigi Ambrosio

**Affiliations:** ^1^Institute for Polymers, Composites and Biomaterials, National Research Council, Mostra d'Oltremare Pad. 20, Viale J. F. Kennedy 54, 80125 Naples, Italy; ^2^Biopharma Commercial, Novozymes Biopharma DK A/S, Krogshoejvej 36, 2880 Bagsvaerd, Denmark; ^3^Department of Chemical Science and Materials Technology DCSMT-CNR, Piazzale Aldo Moro 7, 00185 Rome, Italy

## Abstract

Hyaluronic acid (HA) hydrogels, obtained by cross-linking HA molecules with divinyl sulfone (DVS) based on a simple, reproducible, and safe process that does not employ any organic solvents, were developed. Owing to an innovative preparation method the resulting homogeneous hydrogels do not contain any detectable residual cross-linking agent and are easier to inject through a fine needle. HA hydrogels were characterized in terms of degradation and biological properties, viscoelasticity, injectability, and network structural parameters. They exhibit a rheological behaviour typical of strong gels and show improved viscoelastic properties by increasing HA concentration and decreasing HA/DVS weight ratio. Furthermore, it was demonstrated that processes such as sterilization and extrusion through clinical needles do not imply significant alteration of viscoelastic properties. Both SANS and rheological tests indicated that the cross-links appear to compact the network, resulting in a reduction of the mesh size by increasing the cross-linker amount. In vitro degradation tests of the HA hydrogels demonstrated that these new hydrogels show a good stability against enzymatic degradation, which increases by increasing HA concentration and decreasing HA/DVS weight ratio. Finally, the hydrogels show a good biocompatibility confirmed by in vitro tests.

## 1. Introduction

Hydrogels thanks to their unique properties, such as excellent biocompatibility, high water content, and capacity to degrade into safe products, have attracted great attention and have been extensively used in several biomedical applications such as regenerative medicine, aesthetic medicine, and drug delivery [1–3].

Hyaluronic acid (HA), also referred to as hyaluronan, is a naturally occurring linear polysaccharide composed of repeating disaccharide units of d-glucuronic acid and* N*-acetyl-d-glucosamine linked by *β*-1-3 and *β*-1-4 glycosidic bonds [4–8]. HA is a primary component of the extracellular matrix (ECM) of the human connective tissues. It is an important structural element in the skin and is present in high concentration in the synovial joint fluids, vitreous humor of the eyes, hyaline cartilage, intervertebral disc nucleus pulposus, and umbilical cord [9–13]. Due to its strong hydrophilic character and its high molecular weight in biological tissues, HA exhibits important structural and functional roles in the body [14]. In fact it is involved in several functions in vivo such as lubrication of arthritis joints and control of the viscoelastic properties of soft tissues and in important cell functions such as cell motility, cell matrix adhesion, and cell organization [15–19]. Thanks to its biocompatibility and chemical-physical and biological properties and due to the ease of chemical functionalization, HA has generated increasing interest among researchers and it is already used in several biomedical applications [20–22]. Clinically, HA is used in soft tissue replacement and augmentation and in surgical procedures and diagnostics. It is employed as a diagnostic marker for many disease states including cancer, rheumatoid arthritis, and liver pathologies and as an early marker for impending rejection following organ transplantation. It is also used for supplementation of impaired synovial fluid in arthritic patients, in aesthetic medicine such as dermal fillers, and in soft tissue surgery such as vocal fold augmentation, as scaffold for tissue engineering applications and as a device in several surgical procedures, particularly as antiadhesive following abdominal procedures and as aid in cataract surgery [23, 24].

When HA is in physiological environments, it is subjected to various degradation processes due to hydrolysis and enzymatic hydrolysis by naturally occurring hyaluronidase. In order to control the degradation rate and to improve its mechanical properties different strategies such as cross-linking or conjugation have been used to stabilize HA and obtain a more stable material maintaining at the same time its fundamental properties [7, 9, 25–30]. In the case of cross-linking, HA reacts with a cross-linking agent that is capable of creating covalent bonds between HA chains, whereas compounds grafted onto HA chains are referred to as conjugates.

In light of the above, HA represents an excellent starting biomaterial to obtain suitable structures for the regeneration of natural tissues.

The major objective for the design of HA based hydrogels is to obtain devices with appropriate mechanical properties; in fact they must have mechanical properties simulating those of the ECM of natural tissues, sustaining enzymatic degradation and being sufficient to withstand compressive forces from the surrounding tissues in vivo without deformation or collapse. Moreover, it was recently demonstrated that the mechanical properties of artificial substrates in vitro environment significantly affect cell functions such as adhesion, proliferation, migration, and differentiation [31–33]. Furthermore, in order to use the hydrogels for several biomedical applications it is necessary that they possess superior syringeability properties through pharmaceutical needles. The current challenge is to design hydrogels with good mechanical properties and at the same time a good syringeability.

The cross-linking degree strongly affects the syringeability of the hydrogel; in particular, as the cross-linking density of a gel increases, the distance between the cross-linked segments becomes shorter. When a load is applied, these shorter segments require a greater force to deflect. Thus, increasing the cross-linking density strengthens the overall network, thereby increasing the hardness or stiffness of the gel. However, when the gel comprises all or mostly pendant HA polymer chains, a low cross-linking density network is formed which results in softer gels. Therefore, a low cross-linking degree determines a soft gel, implying a good syringeability and a high in vivo degradation rate of the gel; on the other side, a high cross-linking degree results in an increase of the gel hardness and a less easy syringeability profile, required in order to have gels with good mechanical properties and with an increased residence time.

HA concentration is another parameter that influences significantly the syringeability profile of the hydrogel. In particular, by increasing HA concentration, the hardness or stiffness of the gel increases, so it is necessary to vary opportunely the polymer concentration in order to obtain a hydrogel with specific requirements to be used for biomedical applications.

Furthermore, hydrogel syringeability depends also on the molecular weight distribution of the polymer that strongly affects the homogeneity of the system.

In this frame the aim of this study was to produce HA based hydrogels with improved syringeability profile while maintaining the mechanical properties.

The design of HA hydrogels requires the consideration of many parameters such as HA source, HA concentration, buffer environment for the hydrogel, nature of the cross-linking agent, and cross-linking agent/HA weight ratio. However, regardless of these elements, the purity of the HA raw material and the safety of the cross-linking technology are crucial elements in achieving a hydrogel that can be safely administered to patients.

For these reasons, in this work HA produced by fermentation of the novel, superior, and safe strain* Bacillus subtilis* has been used to produce hydrogels. This production technology affords a HA product with unique advantageous properties such as reproducible molecular weight and easy formulation properties. In addition, the higher purity, including the absence of metal ions, of* Bacillus subtilis*-derived HA compared to the available sources of HA offers the possibility of heat sterilization with minor degradation under given conditions and allows its use with various ingredients without degradation or decrease in viscosity.

Moreover, the hydrogels were produced according to a new method described in [34] using divinyl sulfone (DVS) as the cross-linking agent and based on a simple, reproducible, and safe process that does not employ any organic solvents. Owing to an effective purification step, the resulting homogeneous hydrogels do not contain any detectable residual cross-linking agent. Furthermore, the cross-linking reaction with DVS involves hydroxyl groups of HA molecular backbone, avoiding modification reactions involving the HA carboxyl groups.

In this work we aimed to produce cross-linked HA hydrogels with improved properties, such as higher homogeneity compared to the standard DVS cross-linked HA hydrogels, good mechanical properties, and at the same time an easier syringeability. The hydrogels were characterized in terms of viscoelastic properties as function of HA concentration and HA/DVS weight ratio (w.r.) and network structural parameters using Small Angle Neutron Scattering (SANS) tests. Moreover, hydrogel degradation and biocompatibility in vitro were studied.

## 2. Materials and Methods

### 2.1. Materials

HA used in this work was produced by Novozymes Biopharma A/S by fermentation of the novel, superior, and safe strain* Bacillus subtilis*. Molecular weight of the HA material was in the range of 0.7 to 1.0 MDa as determined by size exclusion chromatography combined with multiangle laser light scattering detection (SEC-MALS).

DVS was obtained from Merck Gmbh and Sigma Aldrich Co. Hyaluronidase (HAase) from bovine testes was purchased from Sigma Aldrich Denmark A/S (ref. H3506). Phosphate buffer saline (PBS) tablets without calcium and magnesium were obtained from MP Biomedicals Inc. (France).

### 2.2. Hydrogel Preparation

HA was cross-linked according to the method described in [34] and here briefly summarized. The method consists of the following steps: (a) preparation of an alkaline solution of HA; (b) addition of DVS to the solution of step (a), whereby HA is cross-linked with the DVS to form a gel; and (c) neutralization and swelling of the gel.

The hydrogels were prepared with two different HA starting concentrations (5% and 6%) and three different HA/DVS weight ratios (2.5 : 1, 5 : 1, and 8 : 1, which correspond to cross-linking degrees of 40, 20, and 12.5 w/w% and 80, 40, and 25 mole%, resp.).

### 2.3. Viscoelastic and Syringeability Properties

Viscoelastic properties of the hydrogels have been evaluated on a rotational rheometer (Gemini, Bohlin Instruments, UK) using a parallel plate geometry (PP30 cell).

Hydrogel was subjected to periodic oscillation in a dynamic experiment (small amplitude frequency sweep tests) to evaluate the dependence of the viscoelastic parameters, such as the elastic and viscous moduli, *G*′ and *G*′′, upon the frequency.

In dynamic experiment the material is subjected to a sinusoidal shear strain:

(1) $$\begin{array}{c} ϒ={ϒ}_{0}\sin\left(\omega t\right), \end{array}$$

where *ϒ*_0_ is the shear strain amplitude, *ω* is the oscillation frequency (which can be also expressed as 2*πf* where *f* is the frequency in Hz), and *t* is the time. The mechanical response, expressed as shear stress *τ* of viscoelastic materials, is intermediate between an ideal pure elastic solid (obeying Hooke's law) and an ideal pure viscous fluid (obeying Newton's law) and therefore is out of phase with respect to the imposed deformation as expressed by

(2) $$\begin{array}{c} \tau =G^{\prime }\left(\omega \right){ϒ}_{0}\sin\left(\omega t\right)+G^{\prime \prime }\left(\omega \right){ϒ}_{0}\cos\left(\omega t\right), \end{array}$$

where *G*′(*ω*) is the shear storage modulus or elastic modulus and *G*′′(*ω*) is the shear loss modulus or viscous modulus.

*G*′ gives information about the elasticity or the energy stored in the material during deformation, whereas *G*′′ describes the viscous character or the energy dissipated as heat. In particular, the elastic modulus gives information about the capability of the sample to sustain load and return in the initial configuration after an imposed stress or deformation [35].

The ratio between the viscous modulus and the elastic modulus is expressed by the loss tangent:

(3) $$\begin{array}{c} \tan\delta =\frac{G^{\prime \prime }}{G^{\prime }}, \end{array}$$

where *δ* is the phase angle.

The loss tangent is a measure of the ratio of energy lost to energy stored in the cyclic deformation. The phase angle, *δ*, is equal to 90° for a purely viscous material, 0° for a pure elastic material, and 0° < *δ* < 90° for viscoelastic materials [36].

The frequency range investigated was 0.01 Hz–1 Hz. In order to identify the linear viscoelastic response range of the materials, preliminary strain sweep tests were performed on the samples, at the oscillation frequency of 1 Hz. The tests were repeated at least three times on each sample. The tests have been carried out at the controlled temperature of 25°C by using a thermostatic bath. In order to avoid water evaporation, the humidity of the chamber containing the samples has been controlled by a humidity control accessory.

In order to evaluate the effect of sterilization process on the viscoelastic parameters, the oscillation tests were repeated on sterile samples, which were obtained by autoclaving at standard conditions (121°C, 15 min).

The effect of injection through needle was evaluated by performing the test on injected samples (gauge lengths 22*G*∗1′′ and 22*G*∗11/2′′, named, resp., (1′′) and (11/2′′)).

The syringeability was measured on a Texture Analyzer (Stable Micro Systems, TA. XT Plus) as the force (in N) needed to inject the hydrogel through a 22*G*1/2 needle over a distance of 55 mm at a speed of 12.5 mm/min (0.2 mL/min) using 1 mL syringes.

The tests were performed on the hydrogels prepared according to the patent [34] (named as new) and on those prepared according to traditional methods and the test was repeated three times.

### 2.4. Small Angle Neutron Scattering (SANS)

The SANS studies were performed on samples characterized by a HA starting concentration of 6% and cross-linker percentage of 1% and 10%, using a LOQ beam line with an ISIS pulsed neutron source. The LOQ beam employs neutrons at wavelengths (*λ*) ranging from 2.2 to 10 Å, which are detected by time-of-flight analysis and recorded with a 64 cm^2^ two-dimensional detector placed at 4.1 m from the sample. Such a setup allows collecting data of scattering vector modulus *Q* = 4*π*/*λ*sin(*θ*/2) in an interval ranging from 0.006 to 0.32 Å^−1^ [37], where *θ* is the scattering angle. The samples studies were contained in 1 mm path length, Hellma quartz cells at 25°C. The experimental raw data were converted on absolute intensity following a previously reported procedure [37].

### 2.5. In Vitro Degradation

In vitro tests were performed to evaluate hydrogel degradation. A stock solution of HAase was prepared from a starting solution of 100 mg/mL of HAase in PBS (43.900 Units/mL). This solution was diluted to a concentration of 43.9 Units/mL and stored at *T* = −20°C prior to use. The hydrogel sample was mixed by vortex with a HAase solution in volume ratio of 10 : 1 to obtain a final enzyme concentration of 4 Units/mL and incubated at *T* = 37°C for different incubation times to analyze the dependence of degradation properties upon time. In order to verify the absence of degradation phenomena due to temperature, a degradation test was carried out on control samples mixed with PBS and incubated at *T* = 37°C. Small amplitude frequency sweep tests were carried out immediately and after 3 h, 9 h, 16 h, and 24 h. The degradation was evaluated by measuring *Gt*′/*G*_0_′ at a frequency of 0.1 Hz as function of time. *G*_0_′ is the *G*′ value just after the mixing with HAase solution at time zero and *Gt*′ is the value of *G*′ at time *t*.

### 2.6. Biological Properties

Mouse embryonic fibroblast NIH3T3 cells were cultured in Dulbecco's modified Eagle's medium supplemented with 10% fetal calf serum (Gibco-BRL Life Technologies, Italy) and antibiotics (penicillin G sodium 100 U/mL, streptomycin 100 g/mL, EuroClone) at 37°C and 5% CO_2_.

For seeding on hydrogels, the cells were washed with phosphate-buffered saline (PBS) and incubated with trypsin-EDTA (0.25% trypsin, 1 mM EDTA, EuroClone), for 5 minutes at 37°C, resuspended in fresh medium, and statically seeded with hydrogel (30.000 cells/cm^2^). The cells were cultured for 1 and 4 days.

Cell viability was evaluated by using Alamar Blue assay. AB was added to the samples (10% v/v of medium) and incubated at 37°C for 4 h. The absorbance of the samples was measured using a spectrophotometer plate reader (Multilabel Counter, 1420 Victor, Perkin Elmer) at 570 nm and 600 nm.

AB is an indicator dye that incorporates an oxidation-reduction indicator that changes colour in response to the chemical reduction in growth medium, resulting from cell viability. Data are expressed as the percentage difference in reduction between treated and control cells in viability assay:

(4) Percentage  difference  between  treated  and  control  cells  =O2×A1−O1×A2O2×P1−O1×P2×100,

where *O*_1_ is the molar extinction coefficient (*E*) of oxidized alamarBlue at 570 nm; *O*_2_ is the *E* of oxidized alamarBlue at 600 nm; *A*_1_ is the absorbance of test wells at 570 nm; *A*_2_ is the absorbance of test wells at 600 nm; *P*_1_ is the absorbance of positive growth control well (cells plus alamarBlue but no hydrogel) at 570 nm; *P*_2_ is the absorbance of positive growth control well at 600 nm.

For proliferation tests, total DNA content of NIH3T3 cells/hydrogel was assessed at 1 and 4 days with Quant-iT PicoGreen dsDNA Reagent Kit. The PicoGreen dye binds to dsDNA and the resulting fluorescence corresponds to the concentration of dsDNA in solution. The total DNA was extracted from each sample by incubating the cell layer in 500 *μ*L of cell lysis solution (0.2% v/v Triton X-100, 10 mM Tris (pH 7.0), and 1 mM EDTA) at 70°C for 1 h, then followed by three cycles of freeze and thaw, and assayed by following manufacturer's instruction (Molecular Probes, Cat. # P-7589). DNA content was determined fluorometrically at excitation wavelength of 480 nm and emission wavelength of 528 nm using a microplate reader (Perkin Elmer Victor microplate reader). The relative fluorescence units were correlated with the number of cells present in the hydrogels.

The osteogenic differentiation of human mesenchymal stem cells (hMSCs) was evaluated by DNA/alkaline phosphatase (ALP) activity measurement. For the DNA/ALP test, at predetermined time point the hydrogels were washed twice with ice-cold PBS, transferred to centrifuge tubes containing 300 mL cell lysis buffer (10 mM Tris-HCL, 10 mM NaH_2_PO_4_/NaHPO_4_, 130 mM NaCl, 1% Triton X-100, and 10 mM sodium pyrophosphate; BD Biosciences), and lysed at −4°C for 45 min. After 5 min of centrifugation, total amount of DNA was detected using PicoGreen Assay (Molecular Probes), while ALP activity was measured using a biochemical assay (SensoLyte pNPP ALP assay kit; ANASPEC).

## 3. Results and Discussion

### 3.1. Hydrogels Viscoelastic and Syringeability Properties

**Figure 1:**
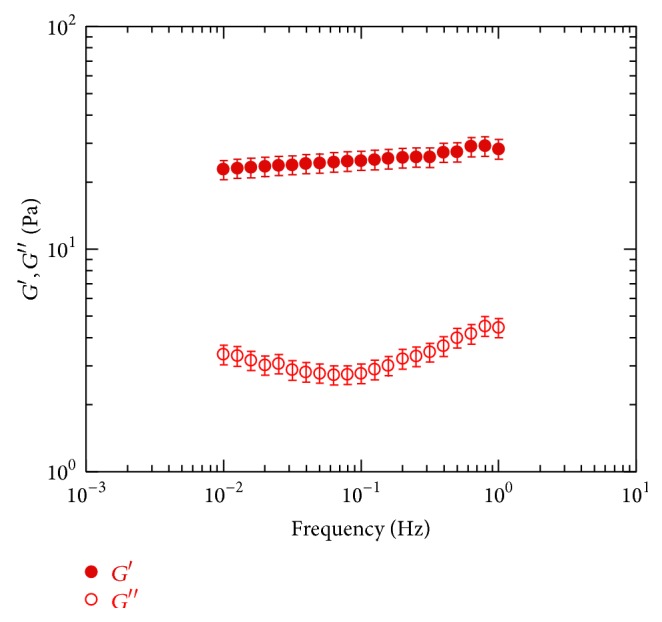
Mechanical spectra of samples characterized by HA/DVS 8 : 1 and [HA] = 5%.

In Figure 1 the dependence of the elastic and the viscous moduli upon the oscillation frequency, the so-called mechanical spectra, for the gels obtained by cross-linking of HA with DVS is reported; in particular, as an example the mechanical spectra of hydrogel with a HA concentration of 5% and a HA/DVS weight ratio of 8 : 1 are reported.

The mechanical spectra show that the elastic modulus is one order of magnitude higher than viscous modulus, *G*′ is almost independent of frequency, and tan⁡*δ* is in the range 0.01–0.1. These samples behave as* strong gel* materials.

The overall rheological response is due to the contributions of cross-links such as covalent bonds and physical cross-links such as electrostatic interactions and hydrogen bonds and chemical and also some topological interactions among the HA macromolecules (entanglements). The cross-links bring about a reduction of the intrinsic mobility of the polymer chains that are not able to release stress; consequently the material shows a predominant elastic behavior (*G*′ > *G*′′) and behaves as a three-dimensional network where the principal mode of accommodation of the applied stress is by network deformation.

Changing HA/DVS weight ratio and HA concentration, the gels still behave as strong gels, but their rheological properties differ quantitatively. In Table 1 the viscoelastic properties for the gels at frequency of 0.1 Hz are reported.

**Figure 2:**
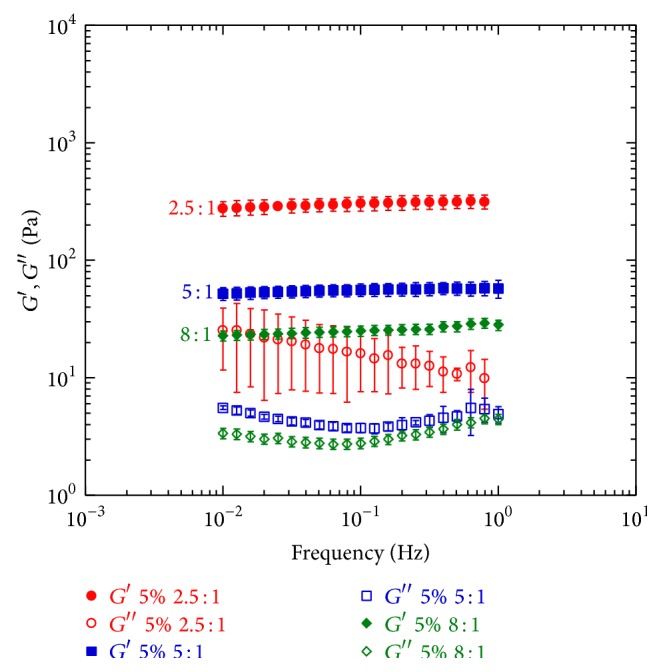
Mechanical spectra of samples at different HA/DVS weight ratio (2.5 : 1; 5 : 1; 8 : 1).

In Figure 2 the mechanical spectra of samples prepared at the same HA concentration (5%) and at different HA/DVS weight ratio (2.5 : 1; 5 : 1; 8 : 1) are reported. Comparison of the results of the three strong gels shows that the highest elastic modulus was obtained in the case of samples characterized by a HA/DVS w.r. of 2.5 : 1. In particular, when HA/DVS w.r. is 2.5 : 1 at a frequency of 0.1 Hz, *G*′ is 304.30 Pa, while for the strong gel at HA/DVS w.r. of 5 : 1 the elastic modulus is 56.20 Pa and for the strong gel at HA/DVS w.r. of 8 : 1 *G*′ is 25.02 Pa (Table 1). By reducing the weight ratio (from 2.5 : 1 to 5 : 1), *G*′ is 5.4 times lower while by decreasing the weight ratio further by 1.6 times (from 5 : 1 to 8 : 1) *G*′ is 2.25 times lower. The increase of the starting HA/DVS weight ratio leads to gels with improved viscoelastic properties because the elastic modulus is proportional to the number of cross-linking points, which increase with the increasing of the amount of cross-linker.

**Figure 3:**
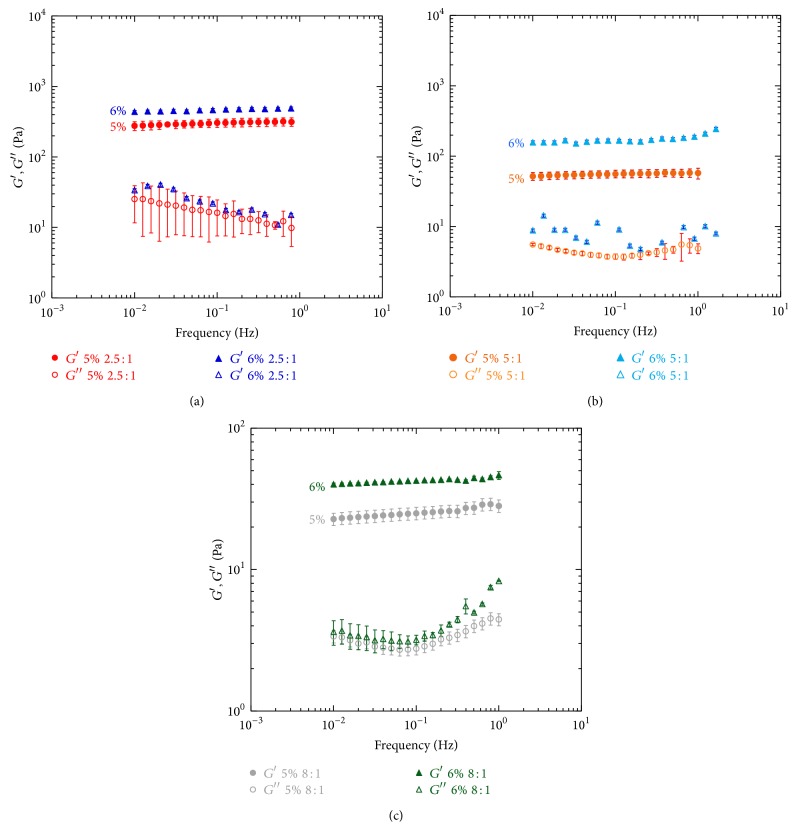
The mechanical spectra of samples at different HA concentrations and HA/DVS weight ratio: (a) the comparison of the mechanical spectra of samples characterized by [HA] = 5% and 6% and HA/DVS weight ratio of 2.5 : 1; (b) the comparison of the mechanical spectra of samples characterized by [HA] = 5% and 6% and HA/DVS weight ratio of 5 : 1; (c) the comparison of the mechanical spectra of samples characterized by [HA] = 5% and 6% and HA/DVS weight ratio of 8 : 1.

Also by varying the starting HA concentration, the viscoelastic properties of the gels change significantly. In particular, the viscoelastic properties increase with increasing of HA starting concentration for any HA/DVS weight ratio (Figures 3(a), 3(b), and 3(c)).

In particular, for samples prepared with HA/DVS w.r. of 2.5 : 1, *G*′ is 304.30 Pa when HA concentration is 5%, while it is 1.5 times higher (468.43 Pa) when HA concentration is 6%. For samples characterized by HA/DVS w.r. of 5 : 1 it can be observed that for HA concentration of 5% *G*′ is 56.2 Pa, while for HA concentration of 6% *G*′ is 165.85 Pa, that is, about 3 times higher. When HA/DVS w.r. is 8 : 1 for HA concentration of 5% *G*′ is 25.02 Pa while for HA concentration of 6% *G*′ is 42,45 Pa, that is, about 1.7 times higher. By decreasing the weight ratio, the effect of concentration becomes stronger. The expected effect of the increase of elastic modulus by increasing the polymer concentration is well known in the literature and was also observed for polyethylene glycol hydrogels [38, 39]. Looking to the viscoelastic properties of HA/DVS gels it can be concluded that, by changing the concentration and HA/DVS w.r., the elastic modulus can be tailored and can increase by about 20 times ranging from about 25 to 470 Pa.

The mechanical properties of these optimized gels are comparable to those of biological gels such as collagen and collagen based network (elasticity of the order of 100 Pa) that are currently used as scaffold for tissue engineering applications or of HA based cross-linked hydrogels used as viscosupplementation product (*G*′ ≈ 80 Pa) [40] or as dermal fillers (*G*′ ≈ 76 Pa) [41] or as augmentation substance for vocal folds regeneration [42]. Moreover, mechanical properties of these optimized gels are similar to chemically cross-linked polysaccharide based hydrogels (*G*′ ≈ 70 Pa) for bone tissue engineering [43] and are in the range of the mechanical properties of many soft tissues, such as the vitreous body (elasticity up to 100 Pa) and the nucleus pulpous (*G*′ ≈ 150 Pa).

To analyze the effect of processing such as sterilization and injection through needle on hydrogels, oscillation tests on sterile and nonsterile samples, before and after injection, were performed.

**Figure 4:**
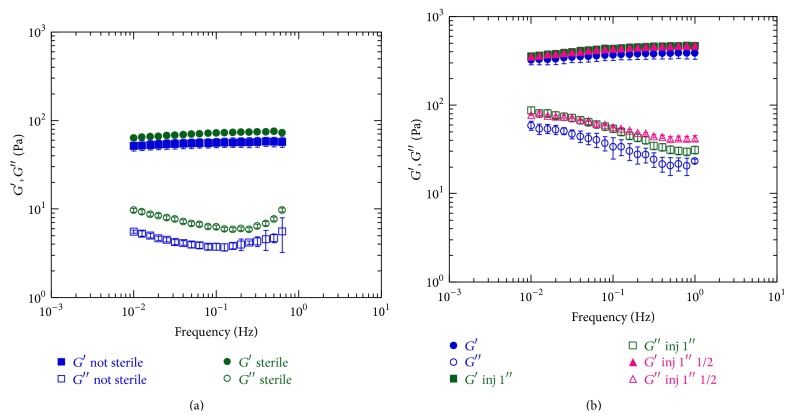
Effect of sterilization and injection on mechanical properties: (a) the comparison of the mechanical spectra of sample characterized by [HA] = 5% and HA/DVS weight ratio of 5 : 1, before and after sterilization; (b) the comparison of the mechanical spectra of samples characterized by [HA] = 5% and HA/DVS weight ratio of 2.5 : 1, before and after injection through different needles.

In Figure 4(a) the comparison between the mechanical spectra of the samples sterilized by autoclave and nonsterile samples is reported. There is no statistical significant difference between the dynamic moduli before and after sterilization, so it can be concluded that the sterilization process does not affect the hydrogel viscoelastic parameters. In Figure 4(b) the mechanical spectra of the sterilized samples are compared before and after the injection through two different needles having different gauge length, *G*∗1′′ (named 1′′) and *G*∗1′′1/2 (named 1′′1/2). It is observed that the injection through the needles does not affect the viscoelastic properties.

**Figure 5:**
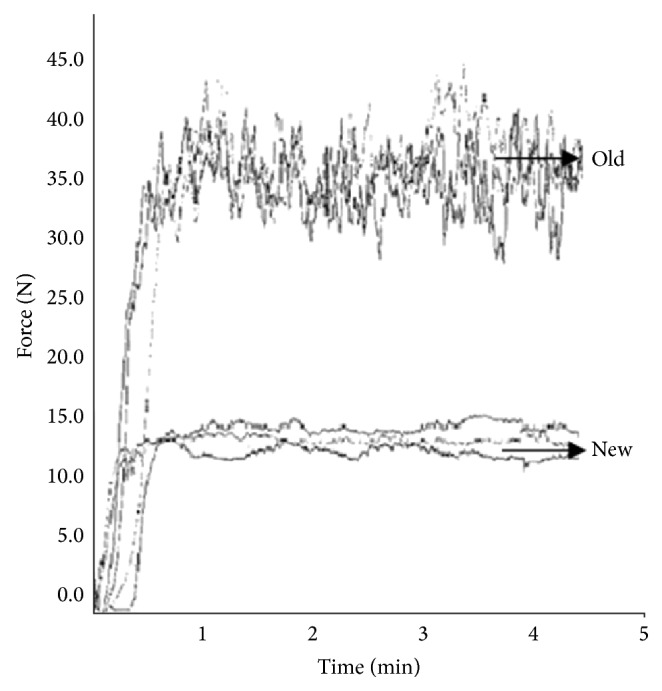
Comparison of syringeability properties of HA/DVS cross-linked hydrogels (new) and hydrogel prepared with traditional methods (old).

In Figure 5 the comparison between the syringeability of the HA/DVS cross-linked hydrogels prepared according the patent [34] and that of the same hydrogels prepared according to traditional methods [44] is shown.

Figure 5 shows that the force needed to inject the HA/DVS cross-linked hydrogels is lower than hydrogels prepared according to traditional methods. Furthermore, injection profile shows that there is a better stability of the applied injection force in the case of the new hydrogels than those prepared according to traditional methods.

Since the stability of injection force is index of the sample homogeneity, the results demonstrated that the new hydrogels were more homogeneous than those obtained with traditional methods and were easier to inject through a fine needle.

### 3.2. Network Structural Parameters

The values of the elastic modulus *G* can be used to estimate the parameter of the network structure [45].

As *G* is proportional to the number of entanglements [36], the elastic modulus can be expressed through

(5) $$\begin{array}{c} G≅R\cdot T\cdot z, \end{array}$$

where *RT* is the thermal energy and *z* is the number of the entanglements points or cross-linking point expressed in mol/volume.

The parameter *z* can be calculated by

(6) $$\begin{array}{c} z\approx \frac{c}{M_{e}}, \end{array}$$

where *c* is the polymer concentration and *M*_*e*_ is the average molecular weight of the polymer segments between two entanglements.

Substituting in (5), *M*_*e*_ can be estimated by the following equation:

(7) $$\begin{array}{c} M_{e}≅\frac{R\cdot T\cdot c}{G}. \end{array}$$

To calculate *G* by means of (7), the validity of the rubber elasticity theory has to be assumed and the temporary network of gel-like material is presumed to behave as vulcanized rubber does upon stimulus of a time scale shorter than the life time of the entanglement network [46].

As the “dangling ends,” which are the polymer chain segments attached to the network by only one entanglement point, do not contribute to the *G* value, because they cannot store elastic energy, a correction is needed in (7) [46]:

(8) $$\begin{array}{c} G≅\frac{R\cdot T\cdot c}{M_{e}}\left(1-2\frac{M_{e}}{M_{n}}\right), \end{array}$$

where *M*_*n*_ is the number average molecular weight.

Using the “equivalent network model” [47], it is possible to give an estimation *D*_*N*_ which is the average distance between the entanglements points or cross-linking points, in an idealized “equivalent network”:

(9) $$\begin{array}{c} D_{N}=\sqrt[{3}]{\frac{6\cdot M_{e}}{\pi \cdot c\cdot A}}, \end{array}$$

where *A* is Avogadro's number.

In Table 2 the results in terms of *D*_*N*_ and *M*_*e*_ are reported. It is observed that the highest *M*_*e*_ (238292 g/mol) and highest *D*_*N*_ (38 nm) are obtained by the sample having the lowest starting concentration and the lowest density of cross-linker (HA concentration of 5% and a HA/DVS w.r. of 8 : 1). Indeed the sample that shows the lowest *M*_*e*_ (61213 g/mol) and a *D*_*N*_ of 24 nm is characterized by the highest starting concentration and the highest cross-linker amount (HA concentration of 6% and a HA/DVS w.r. of 2.5 : 1).

In order to gain deeper structural information on HA/DVS cross-linked hydrogels SANS tests were performed.

**Figure 6:**
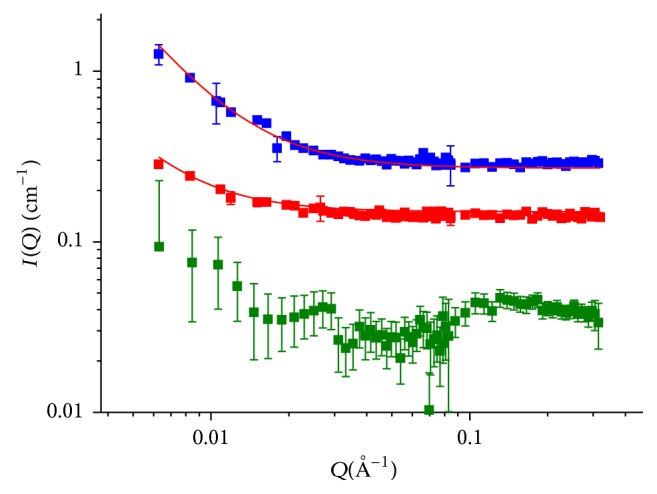
Scattering intensities *I*(*Q*) obtained at 25°C for HA hydrogel samples: green square, uncross-linked HA based solution; red square, hydrogels at HA concentration of 5% and HA/DVS weight ratio of 10 : 1; blue square, hydrogels at HA concentration of 5% and HA/DVS weight ratio of 5 : 1. Lines correspond to fitting of (10) to experimental data.

Figure 6 reports the scattering intensities *I*(*Q*) for hydrogels at HA concentration of 5% and HA/DVS weight ratio of 10 : 1 and 5 : 1; and uncross-linked HA based solution was considered as control.

Analysis of an uncross-linked HA sample reveals a quite flat profile, suggesting a very weak network. Indeed, the scattering should arise from the polymer chains that, because of the low concentration and the high level of solvation, is observed to appear flat. The situation is different in the presence of cross-link. According to the mean-field theory of polymers in a good solvent [48, 49], scattering profile can be described in terms of the mesh size formed by cross-links. It arises from the thermal fluctuations of the polymer concentration and is related to the average distance between cross-links (the mesh size *ξ*): in this case in the region where *qξ* ≪ 1 the scattering intensities *I*(*Q*) can be well described by a Lorentzian function:

(10) $$\begin{array}{c} I\left(Q\right)=\frac{I^{0}}{1+Q^{2}\xi^{2}}, \end{array}$$

where *I* ^0^ is the limit value of the intensity at zero *Q*, related to the number of cross-links per unit of volume.

Equation (10) has been fitted to the experimental data, and the fitting curves are reported in Figure 6 together with the experimental data. From the least square analysis the average mesh size *ξ* has been extracted and reported in Table 3. As it can be observed, the cross-links appear to compact the network, with the mesh size being reduced from about 200 nm to 100 nm when the HA/DVS weight ratio changes from 10 : 1 to 5 : 1. The mesh sizes obtained by SANS tests are in the same order of magnitude of the value of *D*_*N*_ calculated by the rheological tests. Agreement between the values of network structural parameters evaluated by SANS and rheological tests has already observed for cross-linked hydrogels based on other polymers [50].

### 3.3. In Vitro Degradation Properties

**Figure 7:**
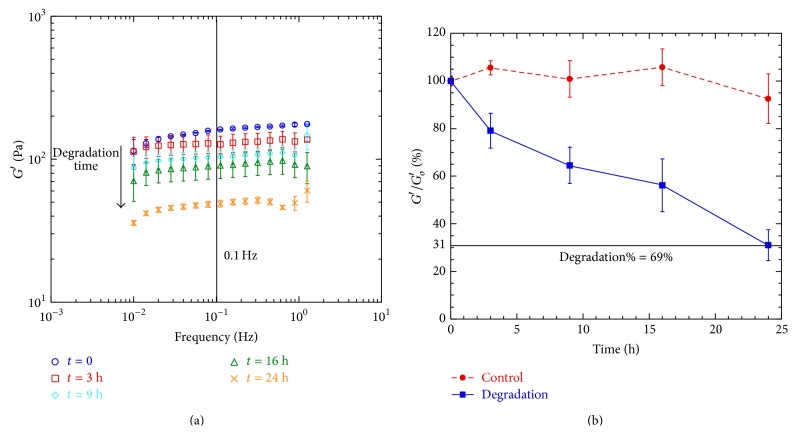
In vitro degradation results: (a) the comparison between the elastic modulus spectra evaluated at different degradation time (0, 3, 9, 16, and 24 h); (b) the evaluation of the percentage *G*′/*G*_0_′ in function of time.

In order to evaluate hydrogels degradation, in vitro tests were performed. In Figures 7(a) and 7(b) the degradation test results are reported. In Figure 7(a) a comparison between the elastic modulus spectra evaluated at different degradation times, for the sample characterized by a HA concentration of 5% and a HA/DVS weight ratio of 5 : 1, is shown. At any frequency, *G*′ decreases with the increase of degradation time, meaning that, in presence of HAase, the degradation occurs.

The evaluation of the ratio *G*′/*G*_0_′ at frequency 0.1 Hz as function of time is reported in Figure 7(b) for one sample. This figure shows a degradation curve and a control curve, where the degradation curve indicates a quantitative evaluation of the percentage of degradation for each time point, and the control curve shows that, in absence of HAase, no degradation was observed. For this sample after 24 h of incubation, the percentage of degradation level is about 70%.

Table 2 shows a summary of degradation tests in which the degradation level for each sample after 24 h of incubation is reported. After 24 h of incubation, no samples show degradation of 100%; the maximum degradation level is about 80%. Furthermore, the samples with the highest amount of cross-linker (HA/DVS w.r. of 2.5 : 1) show a degradation level of 35%.

These results are promising because in the literature it was reported that HA/DVS hydrogels degraded completely in vitro after 24 h and last in vivo up to 6 months [51, 52].

### 3.4. Biological Properties

**Figure 8:**
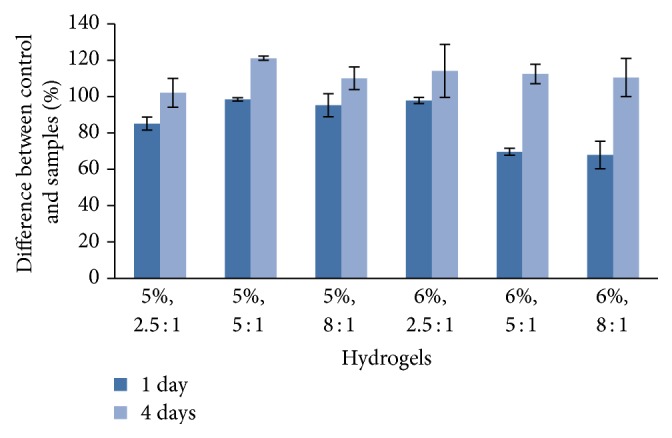
Vitality tests: percentage of reduction of Alamar Blue between hydrogel and controls. The hydrogels are characterized by different HA concentrations (5% and 6%) and different HA/DVS weight ratios (2.5 : 1, 5 : 1, and 8 : 1).

The direct cytotoxicity evaluation results on HA/DVS cross-linked hydrogels were shown in Figure 8. In this graph, the percentage difference in reduction of Alamar Blue between hydrogels and control versus time is reported. The vitality of cells in contact with hydrogels is in the range 70–90% compared to negative control, after 24 hours. After 4 days of contact the vitality of the samples increases up to 100–120%. The assay was stopped at day 4 because the cell proliferation was terminated by contact inhibition.

**Figure 9:**
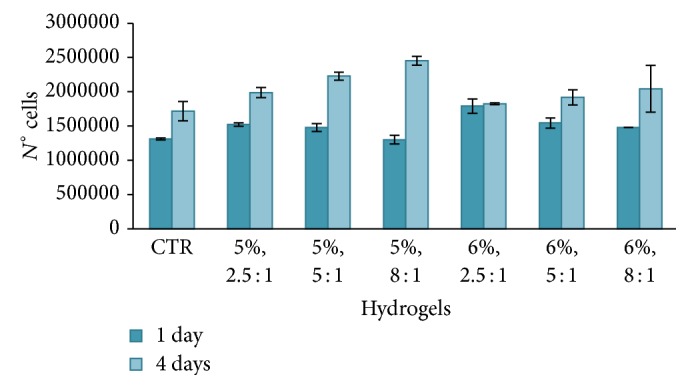
Proliferation tests after 1 and 4 days; the hydrogels are characterized by different HA concentrations (5% and 6%) and different HA/DVS weight ratio (2.5 : 1, 5 : 1, and 8 : 1).

In order to evaluate the proliferation of cells, the results of the PicoGreen test were reported in Figure 9 and expressed as the number of cells alive on the hydrogels after 1 and 4 days.

From Figures 8-9, it can be concluded that the vitality and proliferation of cells placed in contact with the hydrogel are greater than control cells. This behaviour was shown for both HA concentrations, 5% and 6%, and for all considered HA/DVS weight ratios (2.5 : 1, 5 : 1, and 8 : 1). The in vitro tests demonstrate that the hydrogels considered here show a good cellular response and that the cross-linking of HA with DVS is not cytotoxic to cells.

Furthermore, the stem cell differentiation was evaluated by the determination of the ratio between the quantity of ALP and the quantity of DNA; after that the cells were placed in contact with the hydrogels for 7, 14, and 21 days. ALP is the marker that is associated with extracellular matrix development and maturation of the osteoblast phenotypes and is usually upregulated at the earlier stages of osteoblast differentiation [53].

**Figure 10:**
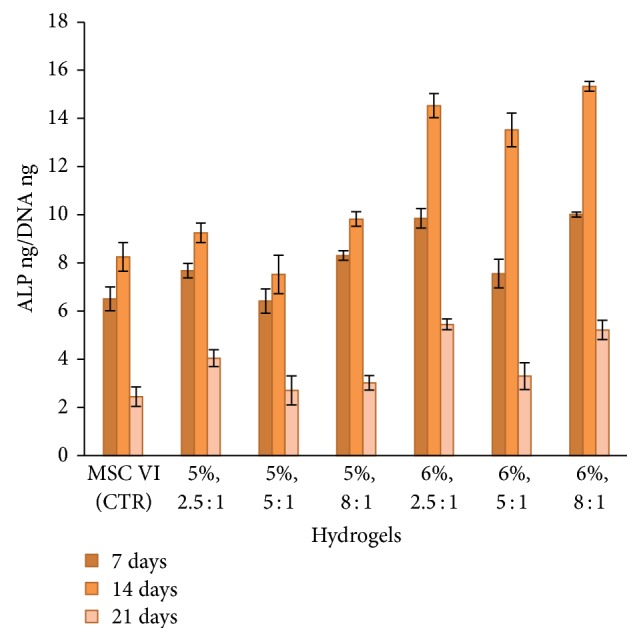
Stem cell differentiation expressed as the ratio between the quantity of ALP and the quantity of DNA; after that the cells were placed in contact with the hydrogels for 7, 14, and 21 days in the presence of osteogenic medium; the hydrogels are characterized by different HA concentrations (5% and 6%) and different HA/DVS weight ratio (2.5 : 1, 5 : 1, and 8 : 1).

The biological test results, reported in Figure 10, show that the ALP activity of mesenchymal stem cells exhibited a typical rise-fall pattern graph for all the samples as it has been reported elsewhere for differentiating osteoblasts [54] in vitro, and in particular the values of ALP ng/DNA ng present a peak after 14 days that the mesenchymal stem cells were put in contact with the hydrogels.

These results suggest that the HA/DVS cross-linked hydrogels, considered in this study, represent good biocompatible environment that do not prevent the stem cells differentiation.

## 4. Conclusion

HA-DVS cross-linked hydrogels based on a simple, reproducible, and safe process that does not employ any organic solvents were developed.

These hydrogels exhibit a mechanical behavior typical of a strong gel and show improved viscoelastic properties by increasing HA concentration and decreasing HA/DVS weight ratio and an improved injectability profile. Furthermore, it was demonstrated that processes such as sterilization and extrusion through clinical needles do not imply significant alteration of viscoelastic properties. The SANS tests demonstrate that the cross-links appear to compact the network, resulting in a reduction of the mesh size by increasing the cross-linker amount. In vitro HA hydrogel degradation tests demonstrated that these new hydrogels show a good stability against enzymatic degradation and a good biocompatibility, confirmed by in vitro tests.

In conclusion these hydrogels show mechanical and injectability properties suitable for regenerative medicine, such as aesthetic medicine (dermal filler applications), and for augmentation substance for vocal folds regeneration and for tissue engineering, such as bone regeneration applications.

## Figures and Tables

**Table 1:** Viscoelastic properties for the gels at frequency of 0.1 Hz.

[HA]	HA/DVS	Viscoelastic properties @ 0.1 Hz			
****[%]	w.r	*G*′ [Pa]	*G*′′ [Pa]	*η* ^∗^ [Pas]	tan⁡*δ*
5	2.5 : 1	304.30	16.10	485.20	0.058
	5 : 1	56.02	3.74	89.36	0.067
	8 : 1	25.02	2.76	40.10	0.12

6	2.5 : 1	468.43	17.7	585.48	0.038
	5 : 1	165.85	9.05	239.17	0.055
	8 : 1	42.45	3.18	67.76	0.075

**Table 2:** Network parameters for HA-DVS cross-linked hydrogels and degradation level after 24 h of incubation with HAase.

[HA]	HA/DVS	*G* ^∗^	*M*_*e*_	*D*_*N*_	Degradation level
[%]	w.r	[Pa]	(g/mol)	(nm)	[%]
5	2.5 : 1	304.30	97104	26	42
	5 : 1	56.02	208107	34	69
	8 : 1	25.02	238292	38	77

6	2.5 : 1	468.43	61213	24	35
	5 : 1	165.85	110457	31	68
	8 : 1	42.45	203958	38	72

^∗^Value of the elastic modulus at 0.1 Hz.

**Table 3:** Mesh size *ξ* obtained for hydrogels at HA concentration of 5% and HA/DVS weight ratio of 10 : 1 and 5 : 1 in D_2_O, estimated from the fitting of (10).

HA concentration, %	HA/DVS weight ratio	*ξ* (nm)
5	10 : 1	223 ± 5
5	5 : 1	94 ± 6

## References

[1] Hoffman A. S. (2012). Hydrogels for biomedical applications. *Advanced Drug Delivery Reviews*.

[2] Borzacchiello A., Mayol L., Ambrosio L., Gärskog O., Dahlqvist Å. (2004). Rheological characterization of vocal folds after injection augmentation in a preliminary animal study. *Journal of Bioactive and Compatible Polymers*.

[3] Jia X., Yeo Y., Clifton R. J. (2006). Hyaluronic acid-based microgels and microgel networks for vocal fold regeneration. *Biomacromolecules*.

[4] Gatej I., Popa M., Rinaudo M. (2005). Role of the pH on hyaluronan behavior in aqueous solution. *Biomacromolecules*.

[5] Lapcık L., de Smedt S., Demeester J., Chabrecek P. (1998). Hyaluronan: preparation, structure, properties, and applications. *Chemical Reviews*.

[6] Ambrosio L., Borzacchiello A., Netti P. A., Nicolais L. Rheological properties of hyaluronic acid based solutions.

[7] Ambrosio L., Borzacchiello A., Netti P. A., Nicolais L. (1999). Properties of new materials: rheological study on hyaluronic acid and its derivative solutions. *Journal of Macromolecular Science—Pure and Applied Chemistry*.

[8] Barbucci R., Rappuoli R., Borzacchiello A., Ambrosio L. (2000). Synthesis, chemical and rheological characterization of new hyaluronic acid-based hydrogels. *Journal of Biomaterials Science, Polymer Edition*.

[9] Monheit G. D., Coleman K. M. (2006). Hyaluronic acid fillers. *Dermatologic Therapy*.

[10] Borzacchiello A., Netti P. A., Ambrosio L., Nicolais L. (2000). Hyaluronic acid derivatives mimic the rheological properties of vitreous body. *New Frontiers in Medical Sciences: Redefining Hyaluronan*.

[11] Borzacchiello A., Ambrosio L. (2001). Network formation of low molecular weight hyaluronic acid derivatives. *Journal of Biomaterials Science, Polymer Edition*.

[12] Barbucci R., Lamponi S., Borzacchiello A. (2002). Hyaluronic acid hydrogel in the treatment of osteoarthritis. *Biomaterials*.

[13] Xin X., Borzacchiello A., Netti P. A., Ambrosio L., Nicolais L. (2004). Hyaluronic-acid-based semi-interpenetrating materials. *Journal of Biomaterials Science, Polymer Edition*.

[14] Volpi N., Schiller J., Stern R., Šoltés L. (2009). Role, metabolism, chemical modifications and applications of hyaluronan. *Current Medicinal Chemistry*.

[15] Mori M., Yamaguchi M., Sumitomo S., Takai Y. (2004). Hyaluronic-based biomaterials in tissue engineering. *Acta Histochemica et Cytochemica*.

[16] Borzacchiello A., Mayol L., Gärskog O., Dahlqvist Å., Ambrosio L. (2005). Evaluation of injection augmentation treatment of hyaluronic acid based materials on rabbit vocal folds viscoelasticity. *Journal of Materials Science: Materials in Medicine*.

[17] Borzacchiello A., Mayol L., Ramires P. A. (2007). Structural and rheological characterization of hyaluronic acid-based scaffolds for adipose tissue engineering. *Biomaterials*.

[18] Fusco S., Borzacchiello A., Miccio L. (2007). High frequency viscoelastic behaviour of low molecular weight hyaluronic acid water solutions. *Biorheology*.

[19] Borzacchiello A., Mayol L., Schiavinato A., Ambrosio L. (2010). Effect of hyaluronic acid amide derivative on equine synovial fluid viscoelasticity. *Journal of Biomedical Materials Research—Part A*.

[20] Gustafson S. (1998). Hyaluronan in drug delivery. *The Chemistry, Biology and Medical Applications of Hyaluronan and Its Derivatives*.

[21] Kogan G., Šoltés L., Stern R., Gemeiner P. (2007). Hyaluronic acid: a natural biopolymer with a broad range of biomedical and industrial applications. *Biotechnology Letters*.

[22] Hemmrich K., von Heimburg D., Rendchen R., di Bartolo C., Milella E., Pallua N. (2005). Implantation of preadipocyte-loaded hyaluronic acid-based scaffolds into nude mice to evaluate potential for soft tissue engineering. *Biomaterials*.

[23] Kablik J., Monheit G. D., Yu L., Chang G., Gershkovich J. (2009). Comparative physical properties of hyaluronic acid dermal fillers. *Dermatologic Surgery*.

[24] Dahlqvist Å., Gärskog O., Laurent C., Hertegård S., Ambrosio L., Borzacchiello A. (2004). Viscoelasticity of rabbit vocal folds after injection augmentation. *Laryngoscope*.

[25] Price R. D., Berry M. G., Navsaria H. A. (2007). Hyaluronic acid: the scientific and clinical evidence. *Journal of Plastic, Reconstructive & Aesthetic Surgery*.

[26] Matarasso S. L., Carruthers J. D., Jewell M. L. (2006). Consensus recommendations for soft-tissue augmentation with nonanimal stabilized hyaluronic acid (Restylane). *Plastic & Reconstructive Surgery*.

[27] Tezel A., Fredrickson G. H. (2008). The science of hyaluronic acid dermal fillers. *Journal of Cosmetic and Laser Therapy*.

[28] Laurent U. B. G., Reed R. K. (1991). Turnover of hyaluronan in the tissues. *Advanced Drug Delivery Reviews*.

[29] Almond A. (2007). Hyaluronan. *Cellular and Molecular Life Sciences*.

[30] Mayol L., Biondi M., Russo L., Malle B. M., Schwach-Abdellaoui K., Borzacchiello A. (2014). Amphiphilic hyaluronic acid derivatives toward the design of micelles for the sustained delivery of hydrophobic drugs. *Carbohydrate Polymers*.

[31] Hachet E., Van Den Berghe H., Bayma E., Block M. R., Auzély-Velty R. (2012). Design of biomimetic cell-interactive substrates using hyaluronic acid hydrogels with tunable mechanical properties. *Biomacromolecules*.

[32] Discher D. E., Janmey P., Wang Y.-L. (2005). Tissue cells feel and respond to the stiffness of their substrate. *Science*.

[33] Evans N. D., Minelli C., Gentleman E. (2009). Substrate stiffness affects early differentiation events in embryonic stem cells. *European Cells and Materials*.

[34] Longin F., Schwach-Abdellaoui K. Method of cross-linking hyaluronic acid with divinylsulfone.

[35] Ferry J. D. (1970). *Viscoelastic Properties of Polymers*.

[36] D'Errico G., de Lellis M., Mangiapia G. (2008). Structural and mechanical properties of UV-photo-cross-linked poly(N-vinyl-2-pyrrolidone) hydrogels. *Biomacromolecules*.

[37] Heenan R. K., Penfold J., King S. M. (1997). SANS at pulsed neutron sources: present and future prospects. *Journal of Applied Crystallography*.

[38] Lin-Gibson S., Bencherif S., Cooper J. A. (2004). Synthesis and characterization of PEG Dimethacrylates and their hydrogels. *Biomacromolecules*.

[39] Lin-Gibson S., Jones R. L., Washburn N. R., Horkay F. (2005). Structure-property relationships of photopolymerizable poly(ethylene glycol) dimethacrylate hydrogels. *Macromolecules*.

[40] Barbucci R., Lamponi S., Borzacchiello A. (2002). Hyaluronic acid hydrogel in the treatment of osteoarthritis. *Biomaterials*.

[41] Santoro S., Russo L., Argenzio V., Borzacchiello A. (2011). Rheological properties of cross-linked hyaluronic acid dermal fillers. *Journal of Applied Biomaterials and Biomechanics*.

[42] Borzacchiello A., Mayol L., Ambrosio L., Gärskog O., Dahlqvist Å. (2004). Rheological characterization of vocal folds after injection augmentation in a preliminary animal study. *Journal of Bioactive and Compatible Polymers*.

[43] Dessì M., Borzacchiello A., Mohamed T. H. A., Abdel-Fattah W. I., Ambrosio L. (2013). Novel biomimetic thermosensitive *β*-tricalcium phosphate/chitosan-based hydrogels for bone tissue engineering. *Journal of Biomedical Materials Research—Part A*.

[44] Biomatrix

[45] De Smedt S. C., Dekeyser P., Ribitsch V., Lauwers A., Demeester J. (1993). Viscoelastic and transient network properties of hyaluronic acid as a function of the concentration. *Biorheology*.

[46] Flory P. J. (1953). *Principles of Polymer Chemistry*.

[47] Schurz J. (1991). Rheology of polymer solutions of the network type. *Progress in Polymer Science*.

[48] de Gennes P. G. (1979). *Scaling Concepts in Polymer Chemistry*.

[49] Koberstein J. T., Picot C., Benoit H. (1985). Light and neutron scattering studies of excess low-angle scattering in moderately concentrated polystyrene solutions. *Polymer*.

[50] D'Errico G., de Lellis M., Mangiapia G. (2008). Structural and mechanical properties of UV-photo-cross-linked poly(*N*-vinyl-2-pyrrolidone) hydrogels. *Biomacromolecules*.

[51] Burdick J. A., Chung C., Jia X., Randolph M. A., Langer R. (2005). Controlled degradation and mechanical behavior of photopolymerized hyaluronic acid networks. *Biomacromolecules*.

[52] Yeom J., Bhang S. H., Kim B.-S. (2010). Effect of cross-linking reagents for hyaluronic acid hydrogel dermal fillers on tissue augmentation and regeneration. *Bioconjugate Chemistry*.

[53] Bancroft G. N., Sikavitsas V. I., Van Den Dolder J. (2002). Fluid flow increases mineralized matrix deposition in 3D perfusion culture of marrow stromal osteoblasts in a dose-dependent manner. *Proceedings of the National Academy of Sciences of the United States of America*.

[54] Shin H., Zygourakis K., Farach-Carson M. C., Yaszemski M. J., Mikos A. G. (2004). Modulation of differentiation and mineralization of marrow stromal cells cultured on biomimetic hydrogels modified with Arg-Gly-Asp containing peptides. *Journal of Biomedical Materials Research Part A*.

